# Iron oxide nanoparticles with photothermal performance and enhanced nanozyme activity for bacteria-infected wound therapy

**DOI:** 10.1093/rb/rbac041

**Published:** 2022-06-23

**Authors:** Jiaxin Guo, Wenying Wei, Yanan Zhao, Honglian Dai

**Affiliations:** State Key Laboratory of Advanced Technology for Materials Synthesis and Processing, Biomedical Materials and Engineering Research Center of Hubei Province, Wuhan University of Technology, Wuhan 430070, China; State Key Laboratory of Advanced Technology for Materials Synthesis and Processing, Biomedical Materials and Engineering Research Center of Hubei Province, Wuhan University of Technology, Wuhan 430070, China; State Key Laboratory of Advanced Technology for Materials Synthesis and Processing, Biomedical Materials and Engineering Research Center of Hubei Province, Wuhan University of Technology, Wuhan 430070, China; State Key Laboratory of Advanced Technology for Materials Synthesis and Processing, Biomedical Materials and Engineering Research Center of Hubei Province, Wuhan University of Technology, Wuhan 430070, China; Foshan Xianhu Laboratory of the Advanced Energy Science and Technology Guangdong Laboratory, Xianhu Hydrogen Valley, Foshan 528200, China; Shenzhen Research Institute of Wuhan University of Technology, Shenzhen 518000, China

**Keywords:** antibacterial, photothermal therapy, peroxidase-like, catalytic therapy

## Abstract

Metal-based nanomaterials usually have broad-spectrum antibacterial properties, low biological toxicity and no drug resistance due to their intrinsic enzyme-like catalytic properties and external field (magnetic, thermal, acoustic, optical and electrical) responsiveness. Herein, iron oxide (Fe_3_O_4_) nanoparticles (IONPs) synthesized by us have good biosafety, excellent photothermal conversion ability and peroxidase-like catalytic activity, which can be used to construct a photothermal-enzymes combined antibacterial treatment platform. IONPs with peroxide-like catalytic activity can induce H_2_O_2_ to catalyze the production of •OH in a slightly acidic environment, thus achieving certain bactericidal effects and increasing the sensitivity of bacteria to heat. When stimulated by near-infrared light, the photothermal effect could destroy bacterial cell membranes, resulting in cleavage and inactivation of bacterial protein, DNA or RNA. Meanwhile, it can also improve the catalytic activity of peroxidase-like and promote IONPs to catalyze the production of more •OH for killing bacteria. After IONPs synergistic treatment, the antibacterial rate of *Escherichia coli* and *Staphylococcus aureus* reached nearly 100%. It also has an obvious killing effect on bacteria in infected wounds of mice and can effectively promote the healing of *S. aureus*-infected wounds, which has great application potential in clinical anti-infection treatment.

## Graphical Abstract

**Figure:**
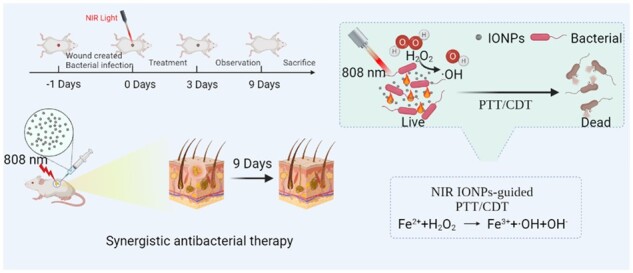

## Introduction

Pathological bacteria are the main cause of bacterial infectious diseases and pose a serious threat to human health [1–3]. At present, the treatment of bacterial infections mainly relies on antibiotics and the long-term abuse of antibiotics has resulted in a sharp decline in the therapeutic effect and serious bacterial resistance [4]. The evolving problem of antimicrobial resistance has led scientific researchers to shift their focus to the ‘post-antibiotic era’, such as metal ions, polymers or antimicrobial peptides (AMPs). Recent studies have found that metal-based nanomaterials usually have intrinsic enzyme-like catalytic properties and external field (magnetic, thermal, acoustic, optical and electrical) responsiveness and can catalyze and induce low concentrations of hydrogen peroxide (H_2_O_2_) transformation into hydroxyl radicals (•OH) [5, 6]. It is a highly cytotoxic reactive oxygen species (ROS) that destroys bacterial cell membranes through oxidation, leading to the inactivation of proteins, DNA or RNA. The unique antibacterial mechanism makes it have a broad-spectrum antibacterial effect and does not induce the generation of bacterial resistance [7–13]. Many nanomaterials with enzyme-like and antibacterial properties have been demonstrated to eradicate different types of bacteria and even drug-resistant bacteria [14–16], including noble metal nanoparticles [17], carbon-based nanomaterials [6, 8], metal oxides [18, 19] and metal chalcogenides [2, 10, 20]; therefore, the development of nanozyme-antibacterial strategies is highly effective and promising [21].

Near-infrared (NIR) light-induced photothermal therapy (PTT) with minimal invasiveness and fewer side effects has received extensive attention in anti-infective therapy [4, 8]. PTT typically utilizes photothermal conversion agents to convert NIR light to hyperthermia, leading to bacterial death by disrupting cell membranes and causing protein denaturation [19]. However, the therapeutic effect of single-mode PTT is limited and can damage healthy tissues due to the high-power density of NIR laser and prolonged irradiation time during treatment [22]. To overcome these limitations, the synergistic treatment of nanozyme-catalyzed therapy and PTT is a promising approach [23, 24]. The combination of catalytic therapy and PTT integrates the advantages of single-modality therapy, which can shorten the antibacterial time and improve the therapeutic effect and enhance the catalytic production of ROS to reduce the indirect damage of PTT [25–27].

In recent years, there have been more and more studies on nanozymes combined with catalysis and photothermal effects for antibacterial therapy. Among the many nanozymes, iron-based nanozymes have excellent photothermal conversion ability and strong enzyme-like catalytic ability in a wide pH range [1, 25, 28, 29]. Iron oxide (Fe_3_O_4_) nanoparticles (IONPs) synthesized by us have good biosafety, excellent photothermal conversion ability and peroxidase-like catalytic activity, which can be used to construct a photothermal-nanozymes combined antibacterial treatment platform. IONPs with peroxide-like catalytic activity can induce H_2_O_2_ to catalyze the production of •OH in a slightly acidic environment, thus achieving certain bactericidal effects and increasing the sensitivity of bacteria to heat. When stimulated by NIR light, IONPs could thermally heat up at the irradiated site. The photothermal effect could destroy bacterial cell membranes, resulting in cleavage and inactivation of bacterial protein, DNA or RNA. Meanwhile, it can also improve the catalytic activity of peroxidase and promote IONPs to produce more •OH for killing bacteria. After IONPs synergistic treatment, the antibacterial rate of *Escherichia coli* and *Staphylococcus aureus* reached nearly 100%. It also has an obvious killing effect on bacteria in infected wounds of mice and can effectively promote the healing of *S. aureus*-infected wounds, which has great application potential in clinical anti-infection treatment.

## Materials and methods

### Materials

Sodium citrate dihydrate, sodium chloride (NaCl), ferric chloride hexahydrate (FeCl_3_·6H_2_O), anionic polyacrylamide (PAM, Mn = 3 000 000), urea and polyethylene glycol-2000 (PEG, Mw = 2000), polyoxymethylene and hydrogen peroxide (H_2_O_2_) were purchased from Sinopharm Chemical Reagent Co., Ltd (China). Dimethyl sulfoxide (DMSO) and 3,3′,5,5′-tetramethylbenzidine (TMB) were purchased from Aladdin Reagent Co., Ltd (Shanghai, China). Fetal bovine serum and Dulbecco’s Modified Eagle Medium were obtained from Gibco (USA). Phosphate-buffered saline (PBS) was purchased from HyClone (USA), Cell Counting Kit (CCK-8) and Calcein-AM/propidium iodide (PI) staining kit was purchased from Shanghai Yeasen Biotechnology Co., Ltd (China). Beef extract, peptone and agar were all purchased from Shanghai Yuanye Biotechnology Co., Ltd (China). *Escherichiacoli* (CICC 10389) and *S. aureus* (CICC 21600) were obtained from the China Center of Industrial Culture Collection (CICC, Wuhan, China).

### Methods

#### Synthesis of IONPs

IONPs were synthesized by the one-step hydrothermal method according to our previous report [30, 31]. Briefly, 2 mmol FeCl_3_·6H_2_O, 4 mmol trisodium citrate trihydrate and 6 mmol urea were dissolved in 40 ml of distilled water, and 2.4 g of PAM and 0.2 g of PEG were added under constant stirring until all ingredients are dissolved and the solution was transparent. The solution was then transferred into a high-pressure autoclave at 220°C for 12 h. The product was separated by a magnet, washed with distilled water and anhydrous ethanol to remove the raw materials with incomplete reaction and finally vacuum-dried and collected.

#### Photothermal effect

Different concentrations (250, 500 and 1000 μg/mL) of IONPs aqueous suspension were added into the EP tube and irradiated with different power densities (1.0, 1.5 and 2.0 W/cm^2^) 808 nm NIR laser. Each group was irradiated for 10 min. The real-time temperatures and thermal images of different samples were recorded using a UTi260B Professional Thermal Imager (UNI-TREND TECHNOLOGY CO., LTD). To determine the photothermal stability of IONPs nanocrystals, the IONPs nanocrystals were cyclically irradiated with NIR light (1.0 W/cm^2^) four on/off cycles for 10 min each time and then cooled to room temperature naturally. The photothermal conversion efficiency of IONPs nanocrystals was calculated by using the methods described in the literature [32].

#### Peroxidase-like activity characterization

The peroxidase-like capacity of IONPs was assessed via the oxidation characteristics of TMB with and without H_2_O_2_ [33]. Briefly, IONPs suspension with a final concentration of 100 μg/mL was prepared in acetic acid-sodium acetate buffer (pH 4.0) and then H_2_O_2_ solution with a final concentration of 1 mmol/L and TMB solution with a final concentration of 1 mmol/L were added at room temperature. The peroxide-like catalytic activity of IONPs was qualitatively evaluated by the color change of the TMB solution [1]. And the OD value of TMB oxidation products was measured by UV–Vis absorbance spectra (OD 652 nm) to investigate its catalytic performance [34]. The effects of temperature and pH value on the activity of IONPs peroxidase were investigated by changing the temperature and pH value of the reaction system during incubation, the temperature range was controlled from 25°C to 50°C and the pH range from 2.0 to 7.0. The photothermal effect of different irradiation time on the peroxide-like activity of the same mixture was studied under the irradiation of 808-nm NIR laser. The UV–Vis absorption spectra of reaction solutions under different irradiation time were recorded. The final catalyst concentration for the kinetics [5] study was 100 μg/mL. Before catalysis, the IONPs aqueous suspension, H_2_O_2_ solution and TMB in DMSO were prepared. The concentration of IONPs was 1000 μg/mL, the concentration range of H_2_O_2_ was 0–40 mM and the concentration range of TMB was 0.1–1 mM. For the kinetics study, 100 μL of IONPs was fully mixed with 700 μL of acetic acid-sodium acetate buffer (pH 4.0) and 100 μL of TMB. The additional 100 μL of H_2_O_2_ solution was added to the above mixture to start the catalysis process. The kinetics were investigated using the Michaelis–Menten equation below

$$V_{0}=V_{\max}\frac{\left[S\right]}{K_{m}+\left[S\right]},$$

where *V*_0_ represents the initial velocity, *V*_max_ refers to the maximum reaction velocity and [*S*] is the concentration of substrate.

#### In vitro *antibacterial experiment*

The antibacterial properties of IONPs were studied by the standard plate counting method. *Escherichiacoli* and *S. aureus* were treated with different conditions. Three parallel samples in each group. The control group was bacterial suspension and treated with H_2_O_2_, NIR or IONPs alone. The monotherapy group was bacteria + IONPs + H_2_O_2_ and bacteria + IONPs + NIR, the synergistic treatment group was bacteria + IONPs + H_2_O_2_ + NIR. IONPs at different concentrations (250, 500 and 1000 μg/mL) were conducted with a series of experiments, the concentration of H_2_O_2_ was 10 mM, the power density of the NIR treatment group was 1.0 W/cm^2^ and the irritation time was 10 min. After different treatments, the bacteria were incubated in a 37°C bacterial incubator for 4 h and 100 μL bacterial suspension was extracted from each experimental group and spread onto the Luria–Bertani (LB) solid plate (LB broth; 1.5% agar) for counting. The viable colonies can be observed after the plates were cultured at 37°C for another 18 h. The relative survival rate of bacteria was calculated according to the following formula (*A*_0_ is the control group, *A*_1_ is the experimental group and the blank control group was denoted as 100%):

$$\mathrm{Antibacterial}=\frac{A_{0}-A_{1}}{A_{0}}\times 100\%.$$

#### Live/dead staining test

The bacteria after different treatments were stained with Calcein-AM and PI for 15 min, followed by washing three times using PBS. Fluorescence microscopy was used to observe the live (green fluorescence) and dead (red fluorescence) bacteria.

#### Morphological characterization of bacteria

IONPs were isolated from bacterial samples treated with different conditions by a magnet and the suspension was centrifuged at 7000 rpm for 5 min. Then the bacteria were dispersed with 4% paraformaldehyde and placed in 4°C refrigerators overnight to fix bacterial morphology. After being washed with PBS three times, the gradient dehydration was carried out with a series of ethanol solutions (10%, 30%, 50%, 70%, 80%, 90% and 100%) successively and each dehydration treatment was 10 min. Finally, the dispersed bacterial suspension was added to the silicon wafer and the morphology of bacteria in each group was observed by scanning electron microscope (SEM, JSM-IT300) after natural air drying and gold spraying.

#### *The eradication of* S. aureus *established biofilm*

The 500 μL *S. aureus* suspension with a concentration of 1 × 10^7^ CFU/mL was added into the 48-well plate or 24-well plate with a cell sliver. Then the plate was placed in a 37°C incubator for 48 h and the LB medium was carefully replaced every 12 h to form biofilm. After 48 h, the supernatant was removed and each sample well was gently washed with PBS to remove unfixed or dead bacteria in the residual medium. Complete *S. aureus* biofilm was formed at the bottom of the well.

The formed biofilms were treated with different conditions, divided into following eight groups: (1) control; (2) PBS + NIR laser; (3) H_2_O_2_; (4) H_2_O_2_ + NIR laser (5) IONPs; (6) IONPs + H_2_O_2_; (7) IONPs + NIR laser and (8) IONPs + H_2_O_2_ + NIR laser. The experimental group (2), (4), (7) and (8) was treated with NIR light (808 nm, 1.0 W/cm^2^) for 10 min. After treatment, the fresh LB medium was added and incubated at 37°C for 12 h. The supernatant was removed and the samples were cleaned with PBS. Crystal violet dye was added to each well for dyeing for 30 min. At the end of dyeing, each well was washed with PBS and the stained biofilm at the bottom of the well was photographed. Then 33% acetic acid solution was added to each well to dissolve crystal violet formed in the well. The absorbance value of the solution at 590 nm was measured with a microplate meter to evaluate the amount of residual biofilm. The biofilm formed on the cell slivers was treated in the above manner, respectively. And SEM was used to observe the morphology of the remaining biofilm as the method described above to evaluate the damage degree of biofilm.

#### In vitro *cytotoxicity assay*

The cytotoxicity of the IONPs was assessed by CCK-8 assay in mouse mononuclear macrophages cells (RAW264.7 cells). RAW264.7 cells were seeded to a 96-well plate (2 × 10^3^ cells each well) and incubated overnight under 5% CO_2_ at 37°C to allow cell adherence. Then the cells were co-cultured with different concentrations of IONPs (0, 20, 50, 100, 200, 500 and 1000 μg/mL) for 1, 3 and 5 day. After incubation, CCK-8 solution (5 mg/mL) was added into each well and incubated for another 2 h. The absorbance of the samples at 450 nm was recorded by microplate reader (Multiskan GO-1510).

#### In vivo *antibacterial activity and wound healing*

All the animal experiments were performed in accordance with the guidelines of the Animal Care and Use Committees of the Wuhan University of Technology. BALB/c mice were randomly divided into following seven groups (*n* = 5): (1) control; (2) PBS + NIR laser; (3) H_2_O_2_; (4) IONPs; (5) IONPs + H_2_O_2_; (6) IONPs + NIR laser and (7) IONPs + H_2_O_2_ + NIR laser. These mice were anesthetized with pentobarbital sodium and then their back was shaved. A skin wound ∼40 mm^2^ was created with sterile surgical scissors. Then, the *S. aureus* suspension (50 μL, 1 × 10^9^ CFU/mL) was gradually inoculated onto the wound and the moisture was allowed to evaporate naturally. After 12 h, for the IONPs + H_2_O_2_ + NIR laser treatment group, 50 μL of IONPs (1000 μg/mL, dispersed into PBS) and 50 μL of H_2_O_2_ (100 μM, dispersed into PBS) were dropped onto the bacterial-infected wound, then the wound was irradiated with a NIR laser (808 nm, 1.0 W/cm^2^) for 10 min. Similarly, the other six groups were given the same procedure. After different treatments, the bodyweight of mice was recorded every other day. At 9 days, the skin samples on mice were collected for histopathological hematoxylin and eosin (H&E) staining and Masson’s trichrome staining analysis. In addition, the mice in each group were dissected and then the main organs (heart, liver, spleen, lung and kidney) were collected for H&E staining to evaluate the biosafety of IONPs.

## Results and discussion

### Photothermal performance of IONPs

**Figure 1.**
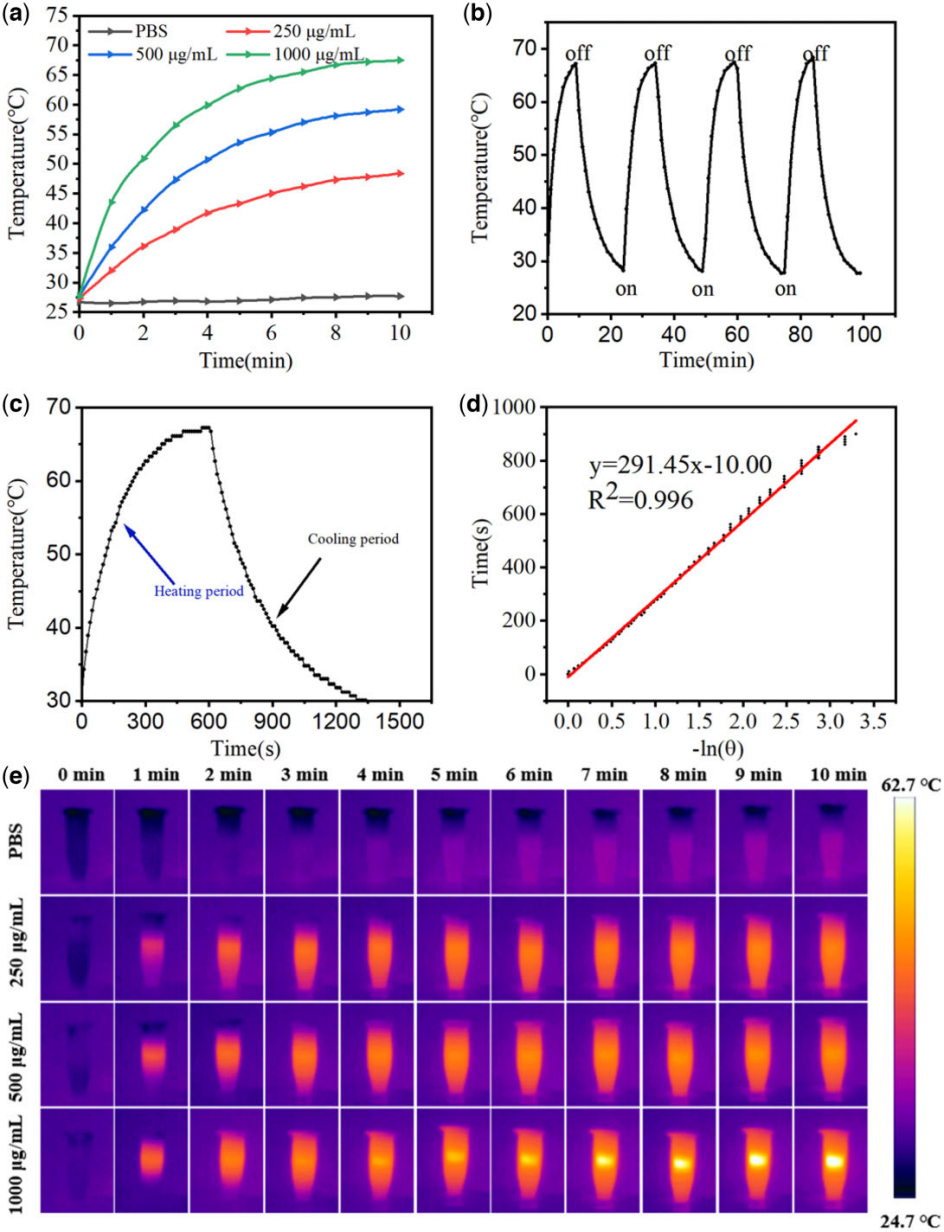
(**a**) Temperatures of different concentrations (250, 500 and 1000 μg/mL) of IONPs suspensions in PBS under NIR irradiation (1.0 W/cm^2^) over 10 min. (**b**) Photothermal heat curves of IONPs (1000 μg/mL) over four NIR irradiation (1.0 W/cm^2^) on/off cycles. (**c**) Temperature variation of primary heating and cooling of IONPs. (**d**) Linear time data versus −ln(*θ*) obtained from the cooling period of (c). (**e**) Real-time infrared thermographic images of different concentrations (0, 250, 500 and 1000 μg/mL) of IONPs suspensions in PBS under NIR irradiation (1.0 W/cm^2^, 10 min).

In order to study the photothermal properties of IONPs suspension, the temperature rise of IONPs  suspension (1000 μg/mL) under different laser power density (0.2, 0.4, 0.6, 0.8 and 1.0 W/cm^2^) was investigated (Supplementary Fig. S2). The temperature curves of IONPs at different concentrations irradiated by 808-nm NIR laser (1.0 W/cm^2^, 10 min) were also investigated. Infrared thermal images and heating curves of IONPs showed that IONPs had good photothermal conversion capability under the irradiation of 808-nm NIR laser and the performance was significantly dependent on concentration, time and laser power, as shown in Fig. 1a and e. The photothermal units required for PTT must have excellent photothermal stability. To this end, we evaluated the cyclic photothermal response capability of IONPs. As shown in Fig. 1b, at NIR light of 1.0 W/cm^2^, after 10 min of continuous cyclic irradiation, IONPs maintained a relatively consistent range of temperature rise and the maximum temperature did not decrease, indicating that IONPs had excellent photothermal stability. In order to analyze the photothermal conversion efficiency of IONPs, we calculated the photothermal conversion efficiency of IONPs of about 28.5% (Fig. 1c and d), which is stronger than that of previously reported Au nanorods (21%), Pt nanoparticles (22.99%), Prussian blue nanocages (26%) and WO_3__−__*x*_ nanoparticles (25.8%) [25]. Therefore, IONPs could be used as a good photothermal conversion agent in the PTT process.

### Investigation of peroxidase-like property

**Figure 2.**
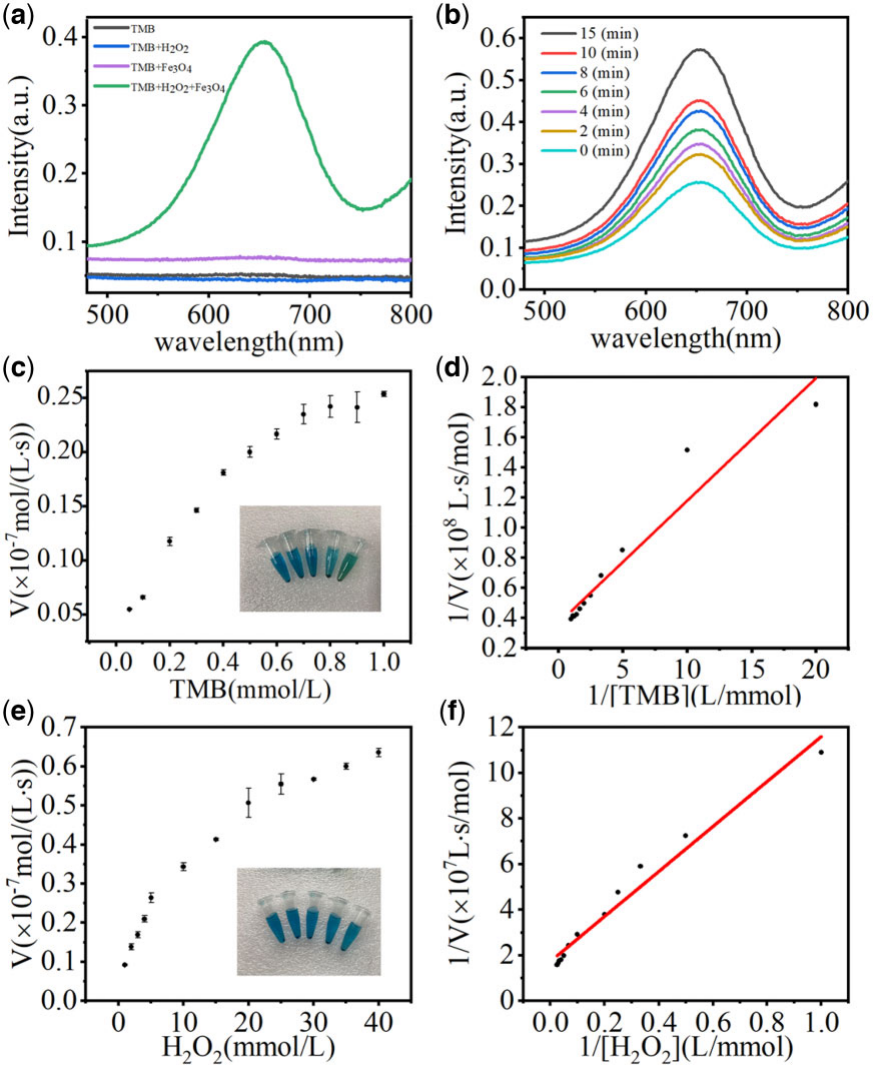
(**a**) UV–Vis absorption spectra of different samples incubated with TMB for 5 min. (**b**) UV–Vis absorbance spectra of TMB (1 mmol/L), IONPs (50 μg/mL) and H_2_O_2_ (1 mmol/L) solution during 15 min with different irradiation time. (**c–f**) Steady-state kinetic assay and catalytic ability of IONPs toward [TMB] (c, d) and [H_2_O_2_] (e, f).

The intrinsic peroxidase-mimicking properties of IONPs were investigated with the catalytic oxidation of TMB and analyzed by UV–Vis spectrometer (Fig. 2a). The control group contained only H_2_O_2_ and TMB. After incubation for 5 min, the absorbance at 652 nm was obvious in the IONPs group, while there was no absorbance in the control group, indicating no peroxidase activity happened. The higher the absorbance intensity, the stronger peroxidase ability was. The peroxidase reaction also caused the color change of the TMB solution, as shown in Fig. 2c. The higher peroxidase-mimicking ability was, the darker blue the solution became, indicating that IONPs have excellent peroxidase-like properties. In addition, the TMB + IONPs + H_2_O_2_ groups were irradiated with different time with 808-nm NIR light, respectively, to explore the effect of irradiation time on peroxidase like activity (Fig. 2b). In the same reaction time, the longer the irradiation time, the more intense the catalytic reaction of the mixture, this is due to the increase of IONPs temperature with time after irradiation by 808-nm NIR laser. Therefore, the peroxide-like properties of IONPs can be significantly improved by irradiating IONPs under an 808-nm NIR laser. The kinetics of the IONPs catalytic process was investigated based on the Michaelis–Menten equation. The Michaelis–Menten constant (*K*_m_) was calculated by the Lineweaver–Burk plot:

$$\frac{1}{V_{0}}=\frac{K_{m}}{V_{\max}}\times \frac{1}{\left[S\right]}+\frac{1}{V_{\max}},$$

where *V*_0_ was gained by the absorption intensity at 652 nm and the molar absorption coefficient of the oxidation product of TMB (39 000/(cm⋅mol/L)) [5]. Based on the above parameters, the Michaelis–Menten curves of IONPs were determined by controlling the concentrations of TMB (Fig. 2c and d) and H_2_O_2_ (Fig. 2e and f), respectively. *K*_m_ and *V*_max_ of IONPs were calculated by the formulation (Table S1). In general, a lower *K*_m_ refers to a stronger attraction between the catalyst and the substrate, while a higher *V*_max_ represents a better catalytic capacity. The whole catalytic process should be assessed in combination with *K*_m_ and *V*_max_. Compared with previously reported data for HRP [35], IONPs showed smaller *K*_m_ values, demonstrating better affinity to TMB and H_2_O_2_ to promote catalytic performance, which makes it a potential peroxidase for action.

Similar to natural enzymes, the effects of pH and temperature on the catalytic reaction of IONPs were also evaluated in this article. As shown in Supplementary Fig. S2a, IONPs showed the best catalytic performance at pH of 2. The relative activity of IONPs decreased with increasing pH, but still exhibited catalytic capacity at pH 7, which was beneficial for the anti-infective therapy *in vitro*. Different from natural enzymes, the catalytic activity of artificial nanozyme increased with the increase of temperature in the range of 25–50°C (Supplementary Fig. S2b).

### *In vitro* antibacterial test

**Figure 3.**
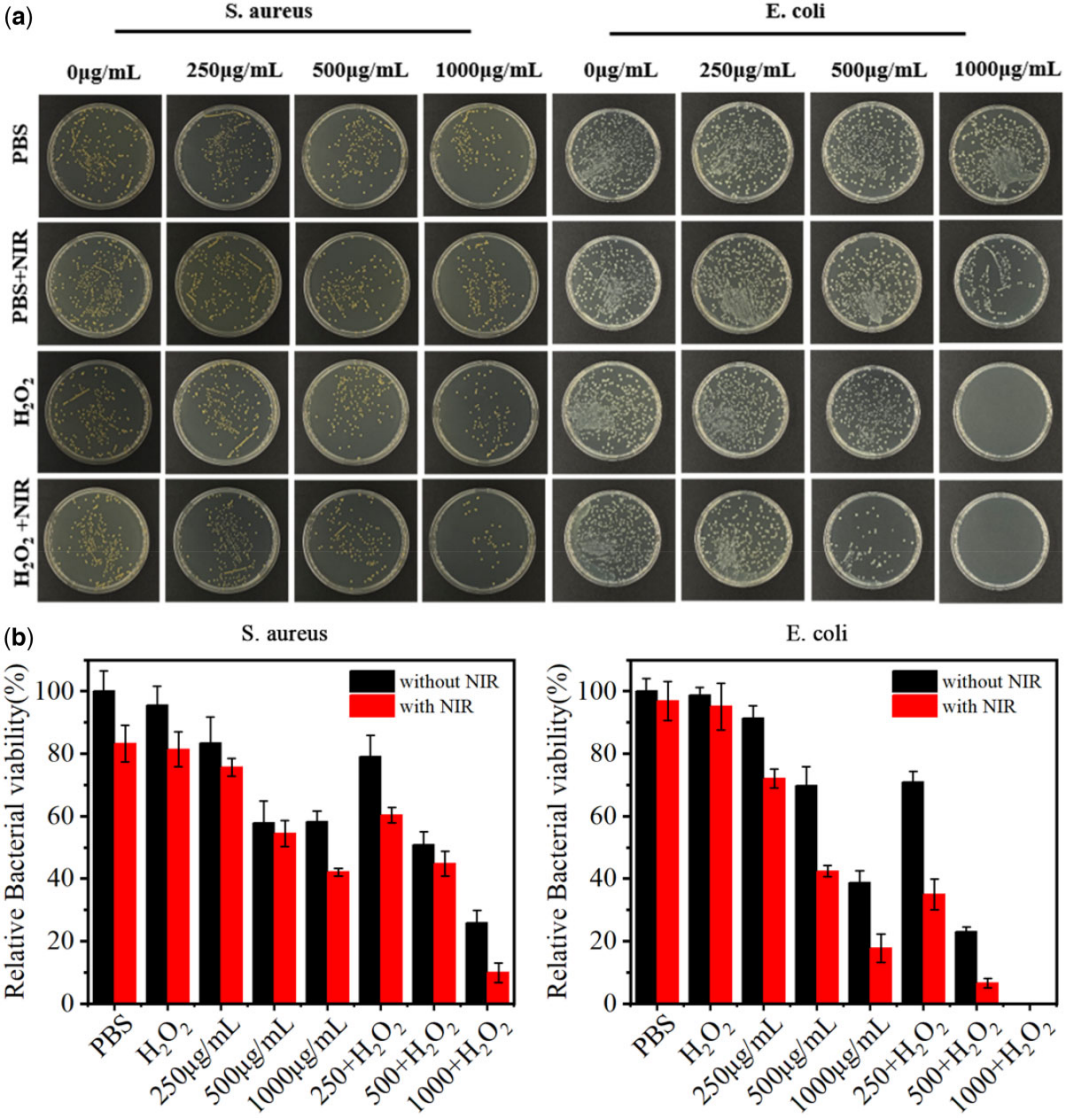
(**a**) Digital photos of viable colonies on standard agar plates cultured with different samples without and with 808 nm NIR laser irradiation. (**b**) Bacteria percentage left in the suspension after removing the catalysts.

Through the above exploration, we found that IONPs had the excellent enzyme-like catalytic ability and photothermal conversion capacity, so we explored the synergistic antibacterial effect of IONPs *in vitro*. First, we investigated the antibacterial effect of IONPs at different concentrations. As shown in Fig. 3a, in the PBS group and the PBS + NIR laser group, a large number of viable bacterial colonies were presented on LB agar plates, showing that NIR laser irradiation alone did not affect the growth of *S. aureus*. In the H_2_O_2_ group and H_2_O_2_ + NIR group, the bactericidal effect of H_2_O_2_ at low concentration is poor and the bactericidal effect was weak regardless of the introduction of NIR irradiation. In the IONPs (1000 μg/mL) treatment group, the colony number decreased by about 41.9%, which was believed to be due to the adsorption of bacteria on the rough surface of IONPs, resulting in the decrease of bacteria in the bacterial suspension after removal of IONPs. When the bacteria suspension was incubated with IONPs and irradiated by the 808-nm NIR laser for 10 min, the number of bacterial colonies number decreased significantly and the survival rate of bacteria decreased from ∼58.1% to ∼42.1%. Apparently, the photothermal ability of IONPs can kill *S. aureus*, but it was insufficient. Compared with the H_2_O_2_ group and IONPs (1000 μg/mL) group, IONPs (1000 μg/mL) + H_2_O_2_ group had a significant antibacterial effect and the bacterial survival rate was 25.9%. These results indicate that IONPs can kill bacteria by catalyzing H_2_O_2_ to toxic ·OH through the Fenton reaction. After NIR laser irradiation, IONPs (1000 μg/mL) + H_2_O_2_ + NIR treatment group had only a small number of bacterial colonies and the antibacterial rate reached 90.1%, suggesting that the synergetic antibacterial platform of the peroxidase-like catalytic and PTT was constructed successfully. With regard to killing *E. coli* (Fig. 3b), the corresponding treatment groups showed the same antibacterial trend as *S. aureus*. However, the *E. coli* was more sensitive to different antibacterial treatments and the bactericidal efficiency of IONPs (1000 μg/mL) + H_2_O_2_ + NIR group reached 100% (Fig. 3b), which may be due to the different membrane structure. The cytoplasmic membrane of Gram-positive bacteria has a thicker peptidoglycan polymer layer, while that of Gram-negative bacteria consists of a thinner peptidoglycan layer [25, 36]. According to the results of the antibacterial performance, and photothermal properties, IONPs with the concentration of 1000 μg/mL was selected for further experiments.

**Figure 4.**
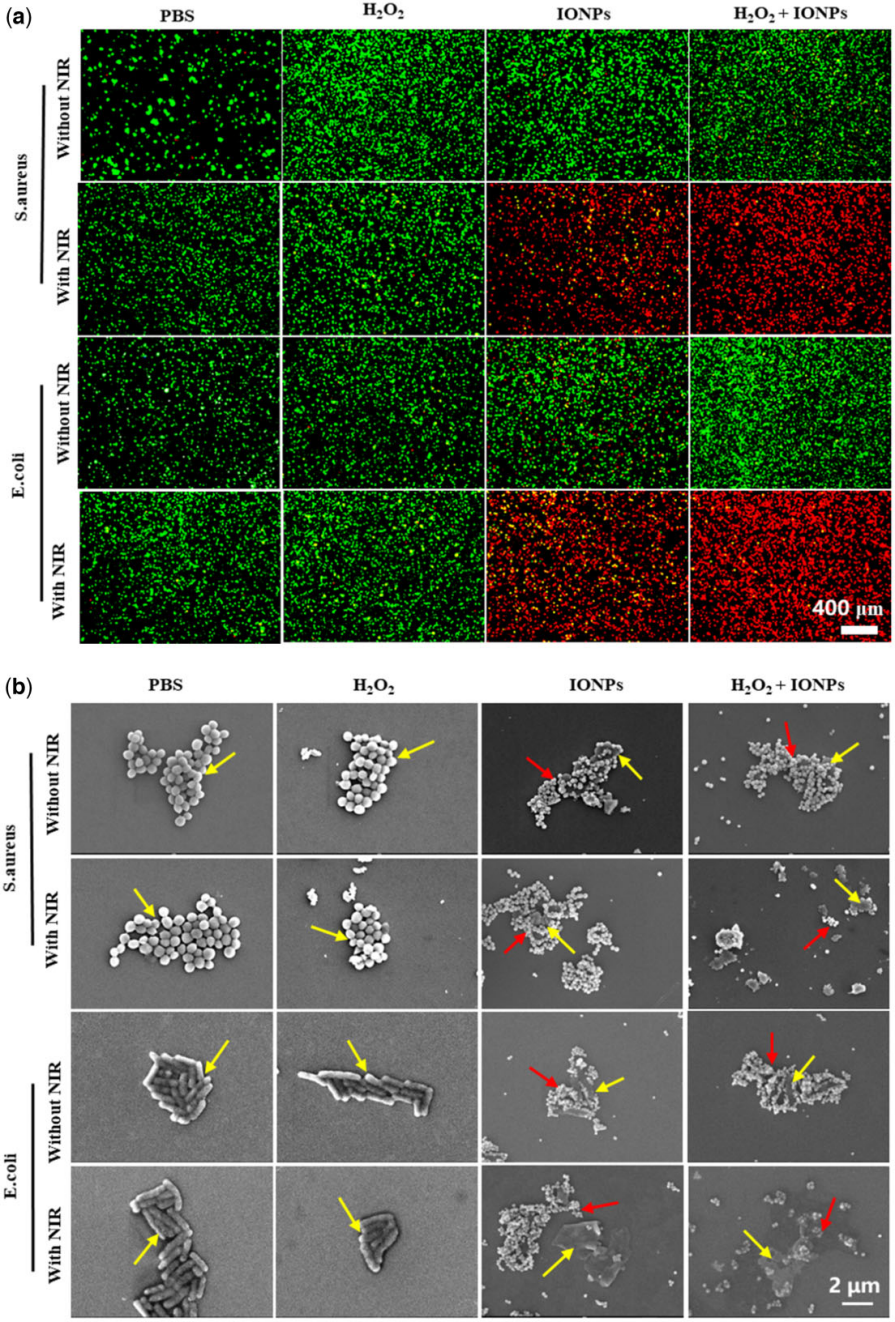
(**a**) The fluorescence images of live/dead bacteria after 808 nm NIR treatment in different groups with calcein–AM staining membrane-complete bacteria and PI staining membrane-damaged bacteria. (**b**) The SEM images show the microstructure of *S. aureus* and *E. coli* in corresponding groups. The scale bars are 2 μm. The yellow arrows indicate the bacterial and the red arrows indicate the IONPs.

To further explain the antibacterial effect *in vitro* described above, the membrane integrity of bacteria was characterized by living/dead bacterial cell staining. Calcein-AM (green fluorescence) was used to stain the live bacteria with the complete cell membrane and PI (red fluorescence) was used to stain the dead bacteria with disrupted membranes. As shown in Fig. 4a, there was no green fluorescence in the IONPs + H_2_O_2_ treatment group under NIR laser irradiation and almost all bacteria were stained with red fluorescence. These results are consistent with the above antibacterial data, which showed that the PTT and peroxidase-like catalytic effect could synergistically eradicate bacteria.

The antibacterial effect of IONPs was also assessed by characterizing the changes in bacterial morphology via SEM. As shown in Fig. 4b, in the PBS group and the PBS + NIR laser group, the bacterial surface was still smooth and intact. When co-incubated with H_2_O_2_ or IONPs for 10 min, the cell membrane surface shows slight distortions and wrinkles, showing that the use of H_2_O_2_ or IONPs alone had little effect on bacterial cell membrane integrity. On the contrary, IONPs + NIR laser treatment group showed obvious cellular deformation and content leakage, indicating that the photothermal effect produced by IONPs damaged the cells. Compared with H_2_O_2_ and IONPs treatment groups, the bacteria in IONPs + H_2_O_2_ showed rougher and more wrinkled membranes, which might be due to the destruction of the cell membrane by ·OH oxidation of H_2_O_2_ catalyzed by IONPs. When NIR irradiation was introduced in the IONPs + H_2_O_2_ group, the bacteria’s cellular membrane integrity was severely damaged and the contents flowed out after 10 min of NIR irradiation, showing an excellent antibacterial effect of the PTT catalytic synergistic therapeutic strategy. Therefore, the antibacterial therapy platform constructed based on the PTT effect and catalysis effect can kill *E. coli* and *S. aureus* quickly and effectively.

### Antibiofilm activity of IONPs *in vitro*

Due to the special composition and structure, extracellular polymer has a strong protective effect on the bacteria encapsulated in the biofilm. It is usually more difficult to eradicate the formed bacterial biofilm than to kill the planktonic bacteria, which is a great challenge for the clinical treatment of biofilm-related infections [37, 38]. The presence of bacterial biofilm is one of the main causes of bacterial drug resistance, leading to many chronic bacterial infection diseases [9, 33]. Therefore, one of the indicators to judge whether antibacterial materials have clinical application value is to evaluate whether they have an excellent ability to destroy biofilms. The main reason for the biofilm infection is usually related to *S.aureus* [22]; thus, we evaluated the effect of IONPs in removing *S. aureus*-established biofilms.

**Figure 5.**
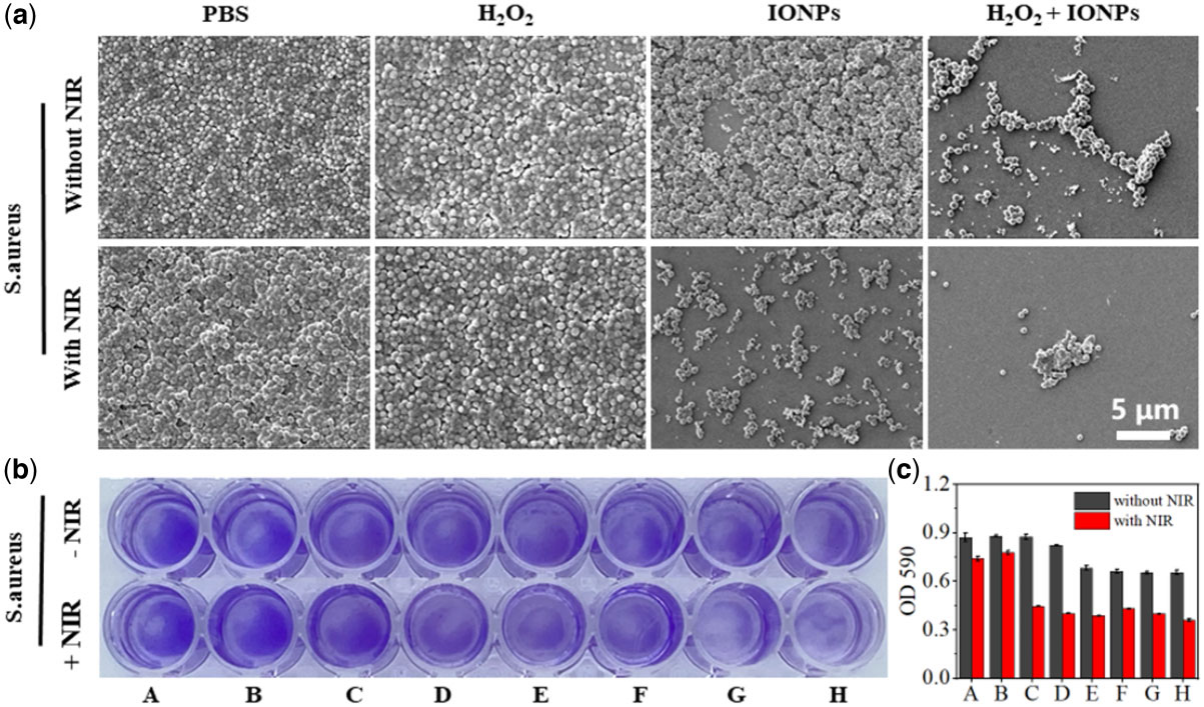
(**a**) SEM and (**b**) crystal violet staining of *S. aureus* biofilm under different combinations with or without exposure to NIR. (**c**) Biomass quantification of biofilms by measurement of absorbance at 590 nm. (A: PBS, B: H_2_O_2_, C: 250 μg/mL IONPs, D: 500 μg/mL IONPs, E: 1000 μg/mL IONPs, F: 250 μg/mL IONPs + H_2_O_2,_ G: 500 μg/mL IONPs + H_2_O_2_, H: 1000 μg/mL IONPs + H_2_O_2_.) The scale bars are 5 μm.

In order to observe the changes of biofilm bacterial morphology more intuitively, the anti-biofilm ability of IONPs was qualitatively analyzed by SEM, as shown in Fig. 5a. The biofilm structure of the control group was complete and tight and the bacterial surface was smooth and intact. After cultured with IONPs, although IONPs can remove some bacterial biofilm, the morphology of bacteria remained intact. However, after adding H_2_O_2_ and irradiation with an NIR laser, the biofilm was damaged completely and the bacteria was distorted and lysed, proving the excellent synergistic antibacterial properties of PTT and catalytic effect of IONPs.

Crystal violet staining (Fig. 5b) showed that 55.7% of the biofilm in the IONPs (1000 μg/mL) + NIR laser group was removed and 58.6% were removed in the IONPs (1000 μg/mL) + H_2_O_2_ + NIR laser combined treatment group compared with that in the control group (Fig. 5c). The results indicated that high temperature and peroxidase-like catalytic effects together incurred the greatest damage against *S. aureus* biofilm, which is consistent with the findings in previous reports that high temperature can destroy the structural integrity of biofilms and subsequently induce the penetration, disruption and dispersal of biofilms by antibacterial agents.

### *In vivo* antibacterial activity and wound healing

**Figure 6.**
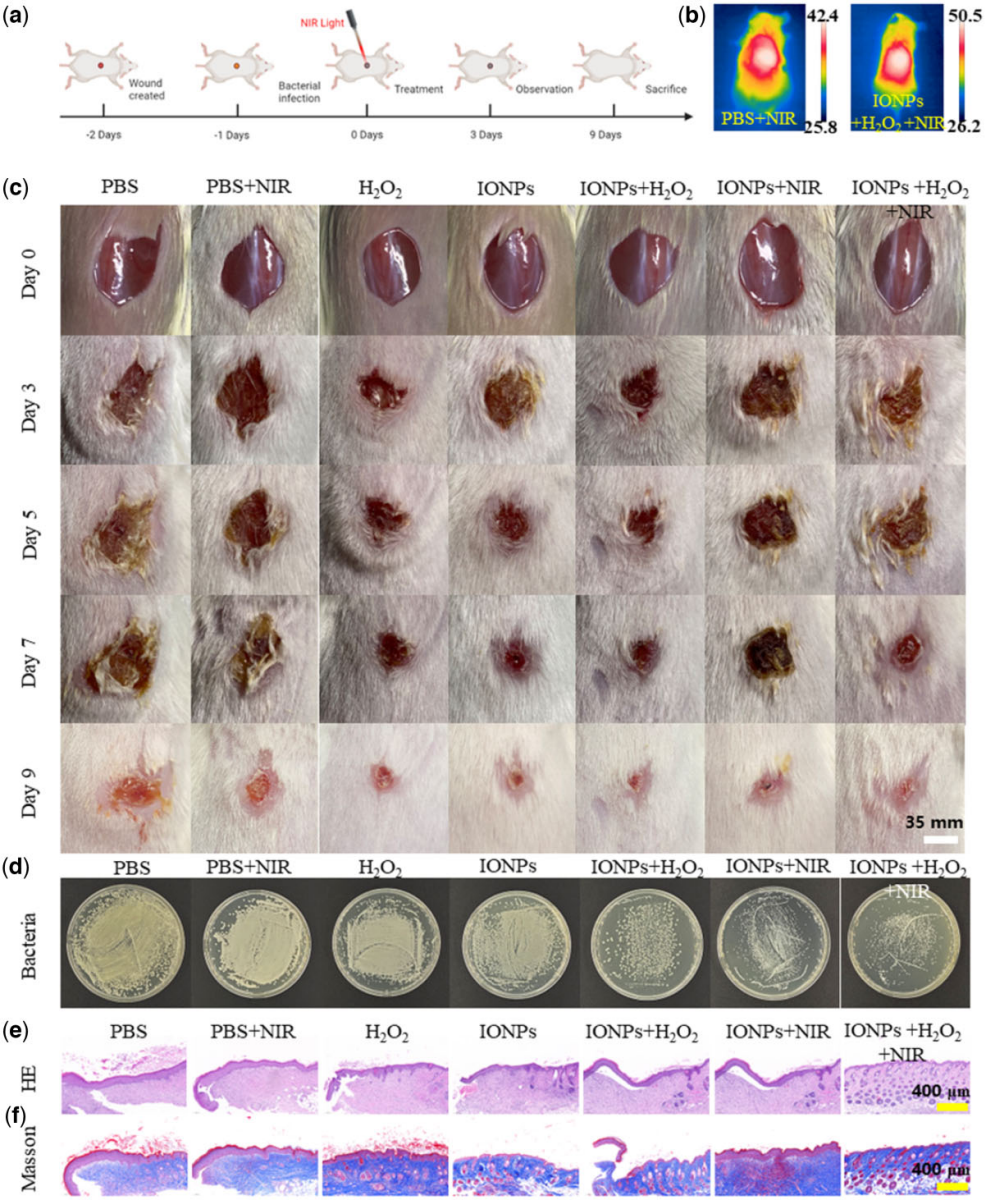
*In vivo* antibacterial performance of IONPs. (**a**) *In vivo* antibacterial protocol in mice. (**b**) Thermal image of mice treated with IONPs after 808-nm laser irradiation (1.0 W/cm^2^, 10 min). (**c**) Wound photographs of mice after various treatments at different treatment time. The scale bars are 35 mm. (**d**) Photographs of bacterial colonies after 9 days treatment. (**e**) H&E and (**f**) Masson staining of the bacteria-infected tissues after different treatments. The scale bars are 400 μm.

We established an animal model of *S. aureus*-infected wound mice to evaluate the *in vivo* antibacterial effect of IONPs, as shown in Fig. 6a. BALB/c mice were randomly grouped and treated under different conditions: (1) control; (2) PBS + NIR laser; (3) H_2_O_2_; (4) IONPs; (5) IONPs + H_2_O_2_; (6) IONPs + NIR laser and (7) IONPs + H_2_O_2_ + NIR laser. During the treatment, H_2_O_2_ concentration was 100 μM and IONPs concentration was 1000 μg/mL. The changes of body weight and wound size of the mice were observed and recorded periodically during the whole treatment process. The IONPs + NIR laser (6) and IONPs + H_2_O_2_ + NIR laser (7) were irradiated by an 808-nm NIR laser with a power density of 1.0 W/cm^2^ for 10 min. The wound temperature of NIR was significantly increased, meeting the expected requirements, while the wound temperature of the control group was slightly increased, which was lower than that of the IONPs group (Fig. 6b). As shown in Fig. 6c, by comparing and analyzing the changes of wound skin of mice in each treatment group, we found that, compared with the control group (1), the relative wound area of groups PBS + NIR laser (2) and H_2_O_2_ (3) did not improve significantly. However, the relative wound area decreased significantly in the IONPs single treatment groups of IONPs + H_2_O_2_ (5) and IONPs + NIR (6). While the wound of the group IONPs + H_2_O_2_ + NIR laser (7) almost completely healed and had the best therapeutic effect. After the treatment, we analyzed the residual bacteria in the skin-infected wound mice by the spread plate method (Fig. 6d). The relevant statistical data analysis of the bacterial colonies was shown in Supplementary Fig. S3. The results showed that the residual bacteria in the control group were the most, while the residual bacteria in group IONPs + H_2_O_2_ (5) and group IONPs + NIR (6) were relatively less than those in the control group, indicating that IONPs single treatment had a certain bactericidal effect. The IONPs + H_2_O_2_ + NIR (7) synergistic treatment group had the least residual bacteria and had the best therapeutic effect compared with other experimental groups. At the same time, the wound skin of mice was observed by HE staining and Masson staining to further evaluate the wound healing effect at the end of treatment (Fig. 6e and f). Significant inflammatory cell infiltration and epidermal disorder were still observed in the control group (1); IONPs + H_2_O_2_ group (5) and IONPs+ NIR group (6) had less wound inflammation, indicating that both IONPs enzyme-like catalytic treatment and photothermal treatment have certain therapeutic effects on bacterial infected wounds. In the IONPs + H_2_O_2_ + NIR group (7), almost no signs of inflammation were observed in the tissue sections and the skin surface structure was intact and orderly, with signs of accessory organ formation. Overall, IONPs combined with photothermal and enzymic catalytic treatment had the best therapeutic effect on infected skin wounds.

### Biosafety evaluation

**Figure 7.**
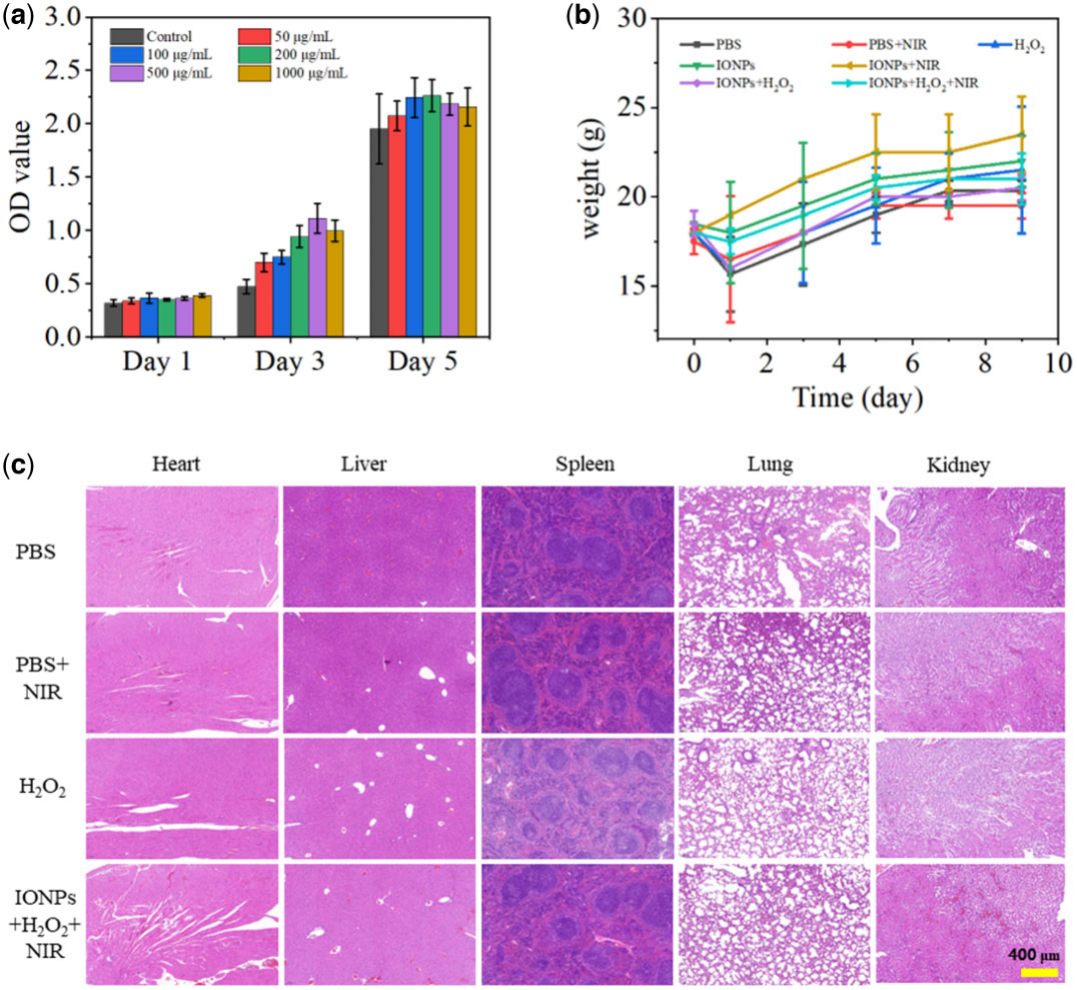
(**a**) Cytotoxicity of different concentrations of IONPs on RAW264.7. (**b**) The body weight changes during various treatments. (**c**) H&E staining images of major organs after different treatments.

The cytotoxicity of IONPs was further evaluated using RAW264.7 cells (Fig. 7a). It was found that the synthesized IONPs had no significant cytotoxicity on RAW264.7 cells, demonstrating the good biocompatibility of IONPs. In order to systematically evaluate the biological safety of IONPs, we recorded and analyzed the changes in the bodyweight of mice during the whole treatment process (Fig. 7b). It can be seen from the results that, consistent with the control group, the body weight of mice in each experimental group increased normally, indicating that IONPs treatment did not affect the growth of mice. After the treatment, we performed H&E staining on the main organs of the heart, liver, spleen, lung and kidney of the mice in each experimental group, as shown in Fig. 7c. Compared with healthy mice, the H&E slices of the main organs of mice in each treatment group did not show any inflammatory response and substantial damage, indicating that IONPs did not have adverse effects on mice during the treatment of infected wounds in mice and showed good biosafety. It is indicated that the IONPs photothermal-nanozyme-catalyzed synergistic antibacterial platform is safe and effective and has great application potential in clinical applications.

## Conclusion

In summary, the synthesized IONPs by us have good biosafety, excellent photothermal conversion ability and peroxidase-like catalytic activity and can be used to construct a photothermal-nanozyme combined antibacterial therapeutic platform. In a slightly acidic environment, IONPs, by virtue of their peroxidase-like catalytic activity, can induce H_2_O_2_ to catalyze the generation of ·OH oxidizing cell membranes, thereby achieving a certain bactericidal effect and improving the sensitivity of bacteria to thermal energy simultaneously. When an external stimulus NIR light is introduced, IONPs can achieve local heating at the NIR irradiation site and the resulting photothermal effect can damage the cell membrane of bacteria, resulting in the cleavage of bacterial proteins, DNA or RNA. The catalytic activity of IONPs promotes the generation of more hydroxyl radicals for killing bacteria. After synergistic treatment with IONPs, the antibacterial rate against *E. coli* and *S. aureus* is close to 100%; it also has a significant killing effect on bacteria in infected wounds in mice and can effectively promote the healing of infected wounds, which has great application potential in clinical anti-infection treatment.

## Funding

This work was supported by grants from the National Natural Science Foundation of China (51772233, 51861145306), the Major Special Project of Technological Innovation of Hubei Province (2019ACA130), the Key Basic Research Program of Shenzhen (JCYJ20200109150218836), Foshan Xianhu Laboratory of the Advanced Energy Science and Technology Guangdong Laboratory (XHT2020-008) and the Fundamental Research Funds for the Central Universities (2020-YB-015).

## Supplementary data

Supplementary data are available at *REGBIO* online.

*Conflicts of interest statement*. None declared.

## Supplementary Material

rbac041_Supplementary_Data
