# Supplementary material for: Iron oxide nanoparticles with photothermal performance and enhanced nanozyme activity for bacteria-infected wound therapy
Source: Regen Biomater. 2022 Jun 23;9:rbac041. doi: 10.1093/rb/rbac041 (PMC9258688; doi:10.1093/rb/rbac041)
Supplement: rbac041_Supplementary_Data [file rbac041_supplementary_data.docx]

**Iron oxide Nanoparticles (IONPs) with photothermal performance and enhanced nanozyme activity for bacteria-infected wound therapy**

Jiaxin Guo ^1^, Wenying Wei ^1^, Yanan Zhao ^1^, Honglian Dai ^1,2, 3, *^

^1^ State Key Laboratory of Advanced Technology for Materials Synthesis and Processing, Biomedical Materials and Engineering Research Center of Hubei Province, Wuhan University of Technology, Wuhan 430070, China

^2^ Foshan Xianhu Laboratory of the Advanced Energy Science and Technology Guangdong Laboratory, Xianhu hydrogen Valley, Foshan 528200, China

^3^ Shenzhen Research Institute of Wuhan University of Technology, Shenzhen 518000, China

* Correspondence address. E-mail: daihonglian@whut.edu.cn

| Catalyst | Substrate | K_m_ (mmol/L) | V_max_ (🞨10^-7^mol/(L·s)) |
| --- | --- | --- | --- |
| MHFP | TMB | 0.22 | 0.28 |
|  | H_2_O_2_ | 5.7 | 0.57 |
| HRP | TMB | 0.43 | 1.00 |
|  | H_2_O_2_ | 3.70 | 0.87 |

Table S1 Comparison of the kinetic data of IONPs with that of HRP in the previous literature.


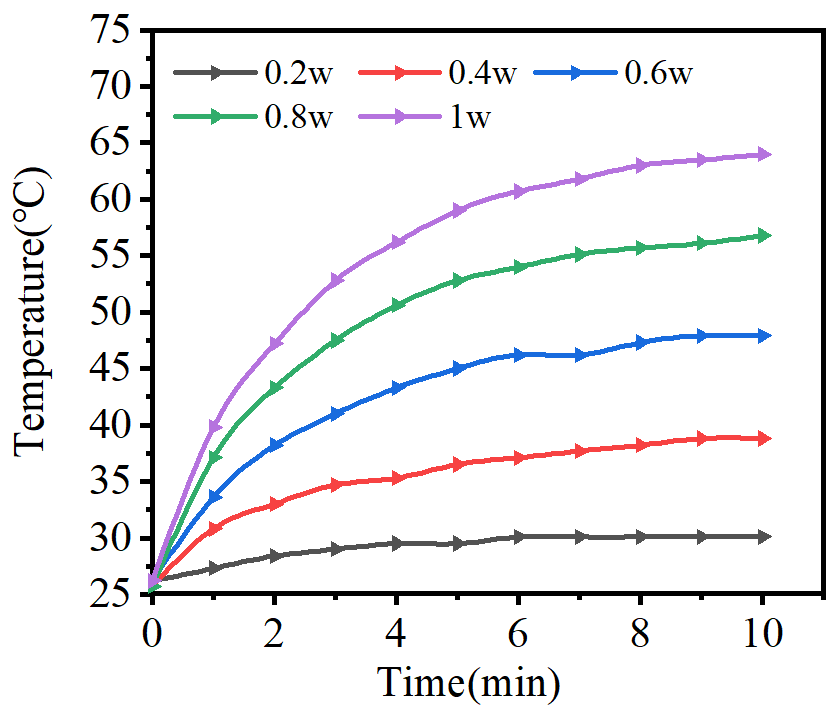


Figure S1. Temperatures of different NIR irradiation power (0.2, 0.4 0.6,0.8,1.0 W/cm^2^) over 10 min.


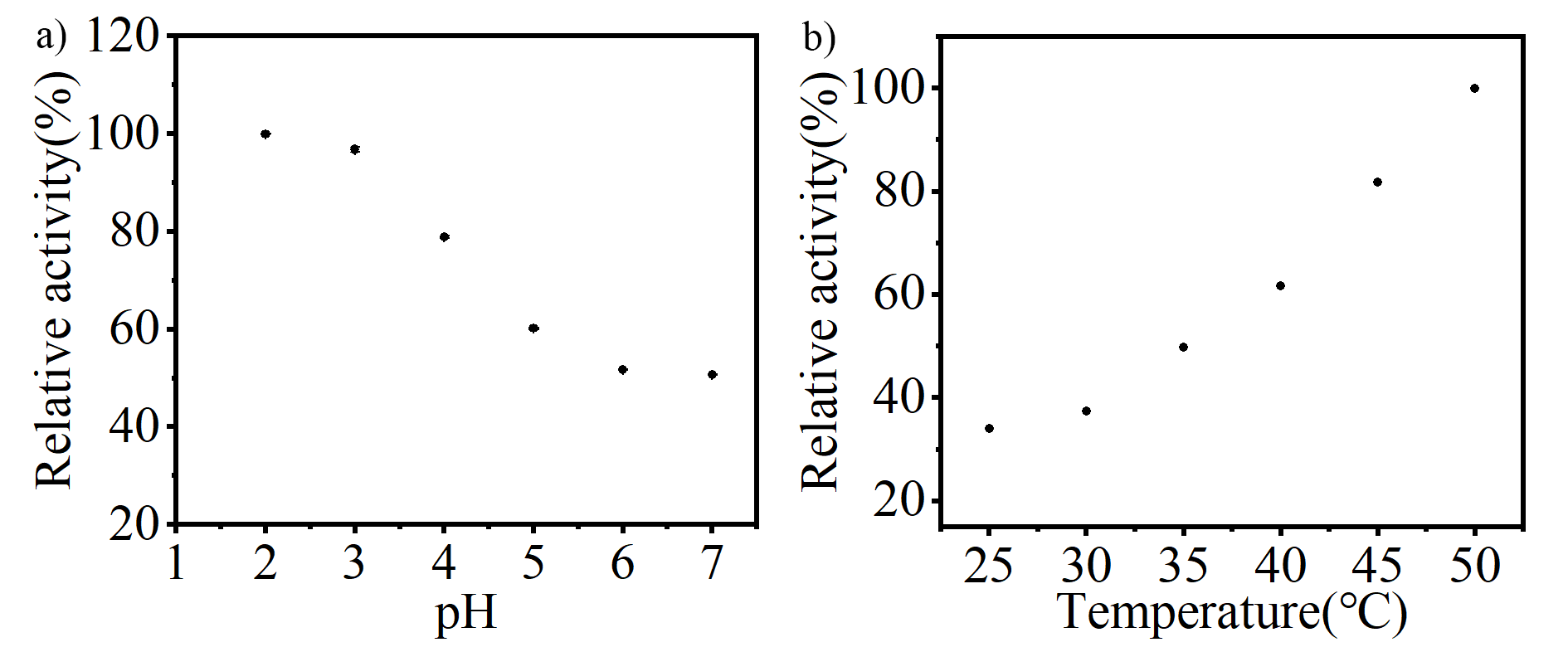


Figure S2. Effects of pH and temperature on the catalytic performance of IONPs.


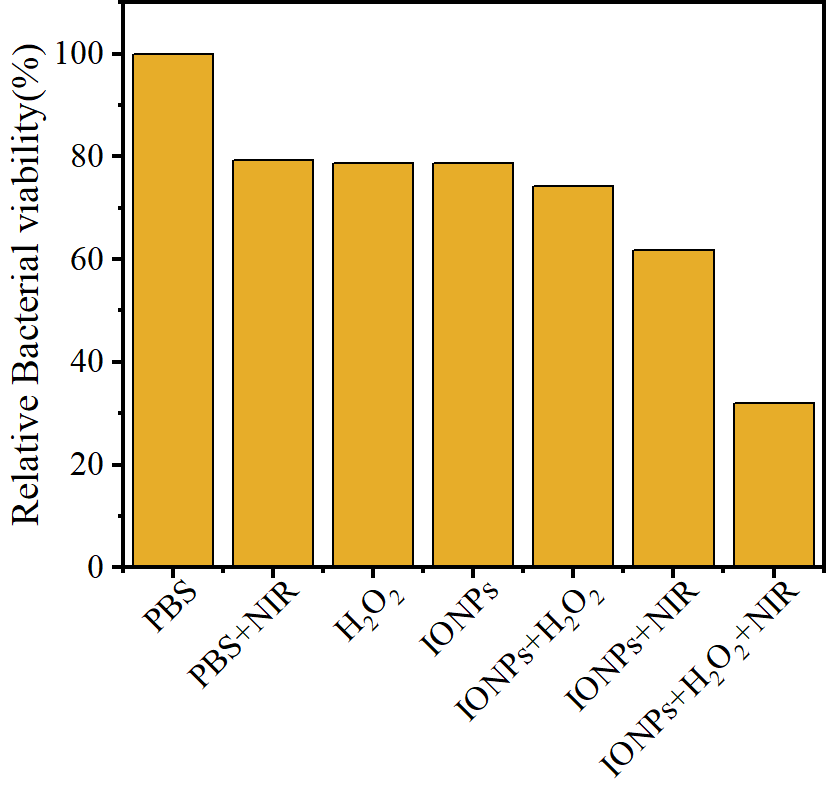


Figure S3. Quantitative analysis of bacterial colonies after 9 days treatment.
